# Oxidative Stress and Biomarkers in Craniofacial Fractures Healing: From Lipid Peroxidation to Antioxidant Therapies

**DOI:** 10.3390/antiox14091070

**Published:** 2025-08-31

**Authors:** Łukasz Woźniak, Żaneta Anna Mierzejewska, Jan Borys, Wioletta Ratajczak-Wrona, Bożena Antonowicz

**Affiliations:** 1 Department of Dental Surgery, Medical University of Bialystok, M. Sklodowskiej-Curie 24A, 15-276 Bialystok, Poland; bozena.antonowicz@umb.edu.pl; 2 Institute of Biomedical Engineering, Faculty of Mechanical Department, Bialystok University of Technology, Wiejska 45C, 15-351 Bialystok, Poland; 3 Department of Maxillofacial and Plastic Surgery, Medical University of Bialystok, M. Sklodowskiej-Curie 24A, 15-276 Bialystok, Poland; jan.borys@umb.edu.pl; 4 Department of Immunology, Medical University of Bialystok, Jerzego Waszyngtona 15A, 15-269 Bialystok, Poland; wioletta.ratajczak-wrona@umb.edu.pl

**Keywords:** oxidative stress, bone healing, biomarkers, craniofacial trauma, antioxidant therapy

## Abstract

Facial bone fractures represent a significant clinical challenge due to their impact on function, aesthetics, and quality of life. Despite advances in imaging and surgical techniques, early and accurate assessment of the healing process remains limited. Conventional diagnostic methods often detect complications, such as delayed union or non-union, too late for optimal intervention. Oxidative stress—an imbalance between reactive oxygen species (ROS) and antioxidant defenses—plays a critical role in bone regeneration. In this review, biomarkers are presented in two main categories: (1) oxidative damage biomarkers (lipid peroxidation products: malondialdehyde, 4-hydroxynonenal, and F2-isoprostanes) and (2) antioxidant biomarkers (glutathione, enzymatic antioxidants: SOD, GPx, CAT). Their potential as non-invasive diagnostic and prognostic tools in craniofacial fracture healing is evaluated, along with emerging therapeutic strategies. Monitoring their levels in blood samples may provide real-time insights into the dynamics of fracture repair, enabling earlier detection of healing disturbances and informing personalized treatment approaches.

## 1. Introduction

Facial bone fractures account for approximately 10–25% of all skeletal injuries, with significant variation depending on geographic region and socioeconomic status. The most common are mandibular fractures (40–60%), followed by midfacial fractures (zygomaticomaxillary complex, orbital wall, naso-orbito-ethmoidal fractures), and Le Fort fractures (I–III) involving the maxilla. Causes vary: traffic accidents (30–50%), interpersonal violence (20–40%), sports injuries (5–15%), and falls (10–20%). Treatment typically involves open reduction and internal fixation (ORIF) with plates and screws, while minor non-displaced fractures may be treated conservatively. Average healing time is 6–12 weeks, though complications such as infection, malunion, and non-union may prolong recovery, especially in smokers, alcohol users, and patients with comorbidities such as diabetes or osteoporosis [1,2,3,4]. In Europe, the most frequent causes are traffic accidents and interpersonal violence, while in Asia occupational accidents and falls dominate the statistics. In children, fractures are most often linked to sports or playground accidents, whereas in the elderly they are frequently associated with falls and osteoporosis-related fragility [1,2,3]. Professional groups at particular risk include construction workers, miners, athletes in contact sports (boxing, martial arts, rugby), and military personnel. Global data also show clear temporal trends: while in the 19th century horse-related accidents dominated, in the 20th century motorization became the leading cause, and in recent decades interpersonal violence and high-energy sports injuries have gained importance. These trends underline the changing profile of patients and the ongoing need for early, accurate diagnostic tools [2,3,4].

Facial bone fractures are among the most complex and impactful injuries in craniofacial trauma, resulting not only in structural and aesthetic impairment but also in considerable functional deficits of the stomatognathic system [1,2,3,4,5]. Despite advances in surgical techniques and radiological imaging, the clinical management of these fractures remains challenging—particularly in monitoring bone regeneration and predicting healing outcomes. Current diagnostic standards, including radiography and CT scans, often detect pathological healing only at advanced stages, limiting opportunities for early intervention [1,6,7,8].

Bone healing is a highly dynamic and tightly regulated biological process involving inflammation, cellular proliferation, callus formation, and remodelling [9]. In recent years, increasing attention has been paid to the role of oxidative stress—defined as the imbalance between reactive oxygen species (ROS) and antioxidant defenses—in modulating these phases. While physiological ROS levels are essential for signalling cascades that initiate bone repair, excessive oxidative stress can disrupt cellular homeostasis, impair osteoblast differentiation, and contribute to delayed or incomplete bone union [10,11,12].

A growing body of evidence suggests that biochemical monitoring of oxidative stress markers may offer a non-invasive window into the molecular status of the healing site [13]. These biomarkers, measurable in blood plasma, have shown promise as early indicators of oxidative imbalance and bone regeneration disturbances [14]. However, their clinical utility remains underexplored due to methodological heterogeneity and a lack of standardized reference values. Oxidative stress biomarkers implicated in bone healing can be classified into two groups: (1) biomarkers of oxidative damage (lipid, protein, and DNA oxidation products), and (2) antioxidant biomarkers (GSH, enzymatic antioxidants, non-enzymatic antioxidants). This review focuses on lipid peroxidation products (MDA, 4-HNE, F2-isoprostanes) and major antioxidant biomarkers (GSH, SOD, GPx, CAT), selected due to their clinical relevance and established detection methods.

The aim of this review was to evaluate the potential of selected oxidative stress markers as diagnostic and prognostic tools in the healing process of facial bone fractures. We pose the question of whether monitoring their levels in the blood can enable early detection of impaired bone union and thereby improve the personalization of treatment.

## 2. Materials and Methods

This narrative review was conducted to synthesize current evidence on the role of oxidative stress biomarkers in the healing of facial bone fractures. Although not registered as a systematic review protocol, the methodology was structured and based on established principles of scoping and semi-systematic reviews.

### 2.1. Data Sources and Search Strategy

This review follows the principles of structured narrative reviews and does not adhere to PRISMA guidelines. A comprehensive literature search was performed using three major biomedical databases: PubMed, Scopus, and Web of Science. The search encompassed articles published between 2000 and 2025. The following keyword combinations were applied using Boolean operators (AND/OR):

- “oxidative stress” AND “bone healing”;
- “biomarkers” AND “facial fracture”;
- “reactive oxygen species” AND “fracture regeneration”;
- “MDA” OR “4-HNE” OR “SOD” OR “GPx” OR “glutathione” AND “bone repair”.

Reference lists of relevant reviews and original studies were also manually screened to identify additional pertinent literature.

### 2.2. Inclusion and Exclusion Criteria

Studies were included in this review if they were original peer-reviewed research articles that investigated the relationship between oxidative stress and bone healing, specifically in the context of craniofacial or maxillofacial fractures. Eligible publications included in vivo or in vitro experimental studies, observational clinical research, and translational studies evaluating biomarkers such as MDA, 4-HNE, SOD, GSH, GPx, CAT, and F2-isoprostanes. Only studies published in English with full-text availability were considered.

Articles were excluded if they consisted of case reports, conference abstracts, opinion papers, or editorials. Furthermore, studies focusing exclusively on systemic oxidative stress unrelated to bone injury or regeneration, as well as those that examined non-craniofacial fracture models without translational value to human physiology, were omitted. Research lacking sufficient methodological detail or appropriate outcome measures was also excluded following critical appraisal.

### 2.3. Study Selection and Data Extraction

The initial search yielded 186 articles across all databases. After removing duplicates and applying the inclusion/exclusion criteria, 137 studies were included in the final review. Titles and abstracts were independently screened by two reviewers. Full-text analysis was conducted to extract information on study design, population (human/animal), type of biomarker assessed, measurement techniques (e.g., HPLC, ELISA, LC-MS/MS), and relevance to bone fracture healing phases.

### 2.4. Methodological Quality Considerations

Although a formal risk of bias assessment (e.g., ROBIS or PRISMA tool) was not applied due to the narrative nature of this review, studies with poor methodology, lack of control groups, or unclear outcome measures were critically appraised and excluded when appropriate.

## 3. Bone Healing Physiology

**Figure 1:**
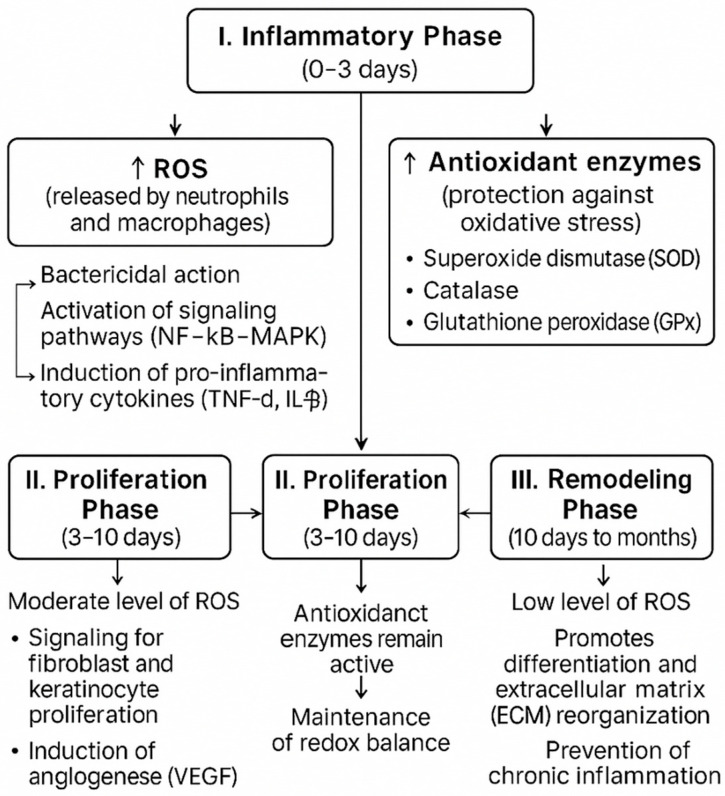
Bone healing physiology.

The process of bone healing after a fracture involves a series of successive cellular and biochemical reactions (Figure 1) triggered by the injury, aimed at restoring the continuity and original structure of the damaged bone. Bone continuity is restored through two possible mechanisms. Primary fracture healing occurs without the formation of a callus, meaning that bone heals through the direct union of the bone fragments [15,16]. This typically happens in clinical conditions where stabilization of the bone fragments is ensured using screws, plates, or nails. The stabilization must be strong enough to prevent micro-movements between the bone fragments, which is critical for primary healing [17].

Secondary fracture healing process begins with the hematoma phase, which is triggered immediately after the fracture. Blood accumulates at the fracture site, forming a hematoma that activates various blood cells, such as macrophages and neutrophils, as well as fibroblasts [18]. These cells release a range of biochemical signals, including cytokines, growth factors, and various mediators are released to stimulate cell proliferation, initiate and regulate the repair mechanisms. These signals attract other cells to the site and begin the process of tissue regeneration. Following this, the organization of the hematoma takes place. In this phase, the hematoma is gradually replaced by inflammatory granulation tissue [18,19,20]. This newly formed tissue serves as a scaffold for the infiltration of cells that are essential for the healing process, such as fibroblasts, endothelial cells, and immune cells. Fibroblasts, or connective tissue cells, produce collagen, which forms the scaffold for the newly forming tissue. During the granulation phase, intense angiogenesis occurs, which is the development of a network of new blood vessels. This is important for delivering nutrients, oxygen, and repair cells to the site of injury. Blood vessels also help remove metabolic waste products and dead cells [21,22]. The next phase is known as cellular metaplasia, during which the granulation tissue undergoes transformation into cartilaginous tissue. This stage marks the beginning of structural restoration, as the soft tissue scaffold becomes more organized and prepared for mineralization. As healing progresses, a temporary callus is formed. This consists of a woven bone structure, also known as immature or primary bone [23,24]. Although this woven bone has relatively low mechanical strength, it plays a crucial role in stabilizing the fracture site and preparing it for the final stage of healing. The final phase is the mature bone formation, or definitive bone union. During this stage, the temporary callus is replaced by a fully mineralized bone matrix. The woven bone is remodeled into lamellar bone, which has a stronger, more organized structure and can withstand normal mechanical stresses [25,26].

The hematoma phase is characterized by the release of pro-inflammatory cytokines such as interleukin-1 (IL-1), tumor necrosis factor alpha (TNF-α), and interleukin-6 (IL-6), which recruit neutrophils and macrophages to the fracture site. These cells clear necrotic tissue and generate reactive oxygen species (ROS), which act as early signalling molecules. Subsequently, vascular endothelial growth factor (VEGF) and fibroblast growth factor (FGF) promote angiogenesis, ensuring oxygen and nutrient supply. In the soft callus stage, chondrocytes produce cartilage matrix, while mesenchymal stem cells (MSCs) differentiate into osteoblasts under the influence of bone morphogenetic proteins (BMPs). During mineralization, osteoblasts deposit hydroxyapatite crystals into the extracellular matrix, whereas osteoclasts orchestrate bone resorption to remodel woven bone into lamellar bone. Transforming growth factor beta (TGF-β) and Wnt/β-catenin signalling pathways coordinate these processes. ROS at physiological levels are essential for these cascades, but excessive oxidative stress impairs osteoblast differentiation, increases osteoclastogenesis, and disrupts the delicate balance of bone turnover.

These phases reflect significant morphological and biochemical changes occurring within and around the fracture site. However, the remodelling process does not stop after this phase but continues throughout life, allowing the bone to adapt to changing mechanical conditions [27].

## 4. Oxidative Stress Mechanisms

In a properly functioning human body, redox homeostasis is delicately maintained, with a slight bias toward oxidants to facilitate essential signalling functions. Reactive oxygen species—including hydrogen peroxide (H_2_O_2_), hydroxyl radical (^•^OH), and superoxide anion (O_2_^•−^)—are predominantly generated during mitochondrial oxidative phosphorylation. These molecules are highly reactive, capable of modifying proteins, lipids, and nucleic acids, which underpins both physiological and pathological processes [27,28,29]. At controlled concentrations, ROS act as critical signalling molecules involved in regulating diverse physiological pathways, such as immune defense and cellular proliferation. For example, ROS stimulate epithelial cell and fibroblast growth, aiding tissue repair [30,31]. However, excessive ROS levels induce oxidative stress, damaging biomacromolecules and precipitating cell death. This oxidative damage is implicated in the pathogenesis of numerous acute and chronic diseases, including impairment of bone healing mechanisms [32]. ROS mediate apoptosis in osteoblasts and osteocytes, thus promoting osteoclastogenesis and disrupting bone remodelling balance. Elevated oxidative stress fosters an inflammatory microenvironment marked by neutrophil infiltration and activation of fibroblasts and osteoclasts, which can delay or impair fracture healing (Table 1) [32,33,34]. This dualistic nature of ROS—being both beneficial and harmful—is highlighted in their immunomodulatory and signalling roles versus their capacity to exacerbate disease states when antioxidant defenses are overwhelmed [35,36].

**Figure 2:**
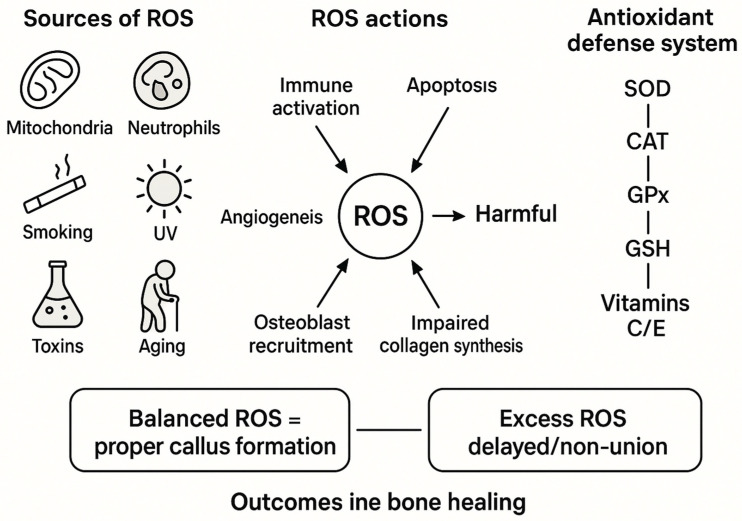
Dual role of ROS in fracture healing.

Oxidative stress results from an imbalance between ROS production and antioxidant capacity. Primary sources of excessive ROS include hyperactivated neutrophils, microbial products, aging, hormonal fluctuations (notably estrogen decline), inflammatory cytokines, environmental toxins, and various drug therapies [37,38]. External factors such as UV radiation, pollution, smoking, and psychological stress further contribute to ROS burden [39]. The body counteracts ROS via enzymatic antioxidants—superoxide dismutase, catalase, glutathione peroxidase, and glutathione reductase—and non-enzymatic antioxidants including vitamins C and E, and glutathione (Figure 2) [40]. These antioxidants are vital in mitigating oxidative damage and facilitating bone regeneration. Recent studies suggest that antioxidant supplementation can enhance fracture healing by protecting osteogenic cells from ROS-induced apoptosis and promoting osteoblast differentiation [41,42]. The initial inflammatory phase of fracture healing is modulated by ROS signalling, which orchestrates cellular recruitment and activation necessary for tissue repair [43,44]. However, chronic oxidative stress prolongs inflammation, delays transition to regeneration, and impairs collagen synthesis critical for extracellular matrix formation. Antioxidants support collagen integrity and vascular endothelial cell function, thus improving microcirculation and nutrient delivery at the fracture site. Furthermore, antioxidants inhibit osteoclast differentiation, tipping the remodelling balance toward bone formation [45,46,47]. Given the complexity of redox regulation in bone healing, there is increasing interest in developing non-invasive methods to monitor oxidative stress biomarkers dynamically during fracture repair. Biochemical assays measuring markers such as malondialdehyde, 8-hydroxy-2′-deoxyguanosine (8-OHdG), and total antioxidant capacity (TAC) in blood samples have shown promise in indicating healing progress and detecting complications early [48,49,50]. Such monitoring enables clinicians to tailor treatments, adjusting antioxidant therapy or employing alternative interventions based on individualized oxidative stress profiles. Clinical trials that have been initiated to investigate antioxidant supplementation, lifestyle modifications, and novel pharmacological agents targeting ROS production to improve bone healing outcomes are also promising [51,52]. Such approaches, combined with marker-based monitoring, have the potential to reduce delayed unions, infections, and malunions, thereby shortening patient recovery time and restoring function. In conclusion, ROS and redox balance play a pivotal, multifaceted role in bone regeneration. While necessary for early signalling, uncontrolled oxidative stress impairs healing. Ongoing research integrating biochemical monitoring and antioxidant-based therapies holds promise for personalized, optimized fracture management strategies.

## 5. Oxidative Stress Biomarkers in Fracture Healing

### 5.1. Oxidative Damage Biomarkers

#### 5.1.1. Malondialdehyde

Malondialdehyde (MDA) is a widely recognized and extensively utilized biomarker of oxidative stress, particularly relevant in the context of bone healing. It is a reactive aldehyde formed as a byproduct of lipid peroxidation (LPO), a free radical-driven oxidative degradation of polyunsaturated fatty acids in cell membranes [53].

MDA is particularly significant due to its high reactivity and ability to form adducts with nucleic acids and proteins, thereby disrupting cellular functions. It exists in biological fluids both as free MDA and covalently bound complexes with biomolecules such as DNA, lipoproteins, and amino acids [54,55]. Elevated MDA levels are indicative of enhanced lipid peroxidation, often reflecting increased production of ROS during pathological and physiological states, including fracture healing [56,57]. The pathological implications of MDA extend beyond its role as a marker. Aldehydes generated during LPO induce DNA strand breaks, exhibit mutagenic and carcinogenic properties, and alter membrane permeability and enzyme function. Additionally, lipid peroxidation products stimulate inflammatory pathways, for instance, by upregulating cyclooxygenase-2 (COX-2) expression in macrophages, which can exacerbate inflammatory responses within the bone microenvironment [58,59,60]. In the context of bone repair, MDA serves as an indicator of the oxidative and inflammatory milieu during fracture healing. The initial inflammatory phase of bone regeneration involves ROS-mediated signalling critical for recruitment and activation of immune cells [44]. However, persistent oxidative stress marked by elevated MDA levels may impair healing by promoting chronic inflammation and osteogenic cell apoptosis [61]. Clinical studies have demonstrated that patients with delayed fracture healing or non-union often present with significantly higher plasma MDA concentrations compared to those with uncomplicated recovery [31,62].

Monitoring MDA levels provides valuable insight into the oxidative status of patients and the efficacy of antioxidant interventions aimed at restoring redox balance during bone healing. Antioxidant therapies, including supplementation with vitamins C and E or glutathione precursors, have been associated with reductions in MDA levels and improved healing outcomes in both experimental models and clinical trials. This supports the premise that controlling lipid peroxidation mitigates oxidative damage and supports the function of osteoblasts and osteoclasts essential for bone remodelling [63,64].

The measurement of MDA is typically conducted using high-performance liquid chromatography (HPLC), which offers high sensitivity and reproducibility, surpassing traditional spectrophotometric assays such as the thiobarbituric acid reactive substances (TBARS) method. Normal plasma MDA levels range between 1.40–1.90 nmol/dL, although these values may vary depending on assay conditions and patient populations [54,65].

Despite the utility of MDA as a biomarker, it is important to consider potential limitations, such as its reactivity and tendency to bind multiple biomolecules, which may complicate quantification and interpretation. Furthermore, oxidative stress is a dynamic process, and MDA levels represent only one facet of redox biology. Combining MDA measurement with other oxidative markers (e.g., 8-isoprostane, protein carbonyls) and antioxidant enzyme activities may provide a more comprehensive assessment of oxidative status during bone healing [65,66].

#### 5.1.2. 4-Hydroxynonenal

4-Hydroxynonenal (4-HNE) is a major toxic aldehydic product generated during non-enzymatic lipid peroxidation, an autocatalytic oxidative process initiated by free radical attacks on polyunsaturated fatty acids (PUFAs) predominantly found in membrane phospholipids. Specifically, 4-HNE is primarily formed through the oxidation of n-6 PUFAs such as linoleic acid, gamma-linolenic acid, and arachidonic acid. The chemical structure of 4-HNE (HOCH_2_-CH=CH-(CH_2_)_4_-CHO) includes three reactive moieties: a hydroxyl group at the C4 position, an aldehyde group at the terminal carbon, and a conjugated double bond between C2 and C3. These reactive groups confer high electrophilicity to 4-HNE, enabling covalent interactions with nucleophilic thiol and amino groups on biomolecules [67,68].

4-HNE’s biological impact is concentration-dependent and involves complex modulation of signalling pathways. At low physiological concentrations (below 5 µM), 4-HNE acts as a signalling molecule, stimulating cell proliferation and mild antioxidant responses through activation of transcription factors such as Nrf2 (nuclear factor erythroid 2-related factor 2), which regulates antioxidant defenses including heme oxygenase-1 (HO-1), glutathione S-transferase (GST), aldehyde dehydrogenases (ALDH), and others [69]. Conversely, elevated levels (20–100 µM) induce cellular stress responses, leading to cell cycle arrest, impaired differentiation, and apoptosis [70]. The threshold for these effects varies by cell type, redox status, and enzymatic capacity for 4-HNE detoxification. Gene expression modulation by 4-HNE includes regulation of NF-kB and AP-1 transcription factors, which orchestrate inflammatory and stress responses. Moreover, depletion of physiological 4-HNE levels has been shown to alter the expression of critical genes such as Fas, Tp53, p21, c-myc, and connexin 43, indicating its role in maintaining cellular homeostasis under normal metabolic conditions [71,72].

Measurement of 4-HNE in vivo remains challenging due to its high reactivity and transient nature. Analytical techniques often detect stable metabolites, or protein adducts rather than free 4-HNE, complicating precise quantification [69,70]. Despite this limitation, elevated 4-HNE adduct levels serve as robust biomarkers of oxidative stress and cellular damage in various pathological states [73].

During bone healing, oxidative stress and inflammation are pivotal in the early phases, facilitating clearance of necrotic tissue and recruitment of progenitor cells [44]. However, excessive accumulation of 4-HNE can prolong inflammation and disrupt normal healing. Studies have demonstrated that elevated 4-HNE impairs osteoblast differentiation and function by modifying signalling pathways and protein activities essential for bone matrix synthesis [33,56]. Additionally, 4-HNE-induced apoptosis of osteogenic cells and mesenchymal stem cells further compromises regeneration [74]. Research by Xiao et al. (2023) [73] showed that oxidative stress-mediated lipid peroxidation products, including 4-HNE, modulate osteoclastogenesis and osteoblastogenesis, suggesting a dual role in bone remodelling. Furthermore, clinical observations link increased systemic 4-HNE levels with delayed fracture healing and non-union, highlighting its potential as a prognostic biomarker.

The dual nature of 4-HNE as both a signalling molecule and a cytotoxic agent underscores the need for tightly regulated redox homeostasis during bone regeneration. While low 4-HNE concentrations activate protective antioxidant pathways via Nrf2, chronic or excessive oxidative stress overwhelms detoxification mechanisms, leading to deleterious modifications in bone-forming cells. Antioxidant therapies targeting lipid peroxidation and 4-HNE scavenging have shown promise in preclinical models to enhance fracture healing outcomes [75,76]. Given the difficulty in directly quantifying free 4-HNE, the detection of 4-HNE-protein adducts via immunochemical methods or mass spectrometry provides a useful proxy for assessing oxidative damage in bone tissues [77].

#### 5.1.3. F2-Isoprostanes (F2-IsoPs)

Discovered in 1990 by Morrow and Roberts, F2-isoprostanes (F2-IsoPs) are prostaglandin F2-like compounds formed by the non-enzymatic, free radical-induced peroxidation of arachidonic acid esterified within phospholipids and lipoproteins [78]. Unlike prostaglandins produced enzymatically via cyclooxygenase, F2-isoprostanes arise independently of enzyme activity, making them highly specific markers of oxidative stress-mediated lipid peroxidation [79].

F2-isoprostanes are considered superior to many other oxidative stress biomarkers because of their stability, sensitivity, and specificity. They can be reliably measured in plasma, urine, and cerebrospinal fluid, facilitating non-invasive assessment of oxidative damage in clinical and experimental settings. Initially generated in an esterified form within membrane phospholipids, F2-isoprostanes are subsequently released as free acids by phospholipases. Their presence in normal tissues and fluids allows for the establishment of baseline reference values, enabling detection of subtle increases indicative of mild oxidative stress [80,81,82].

The biochemical mechanism of F2-isoprostane formation involves the abstraction of hydrogen atoms from arachidonic acid by reactive oxygen species, yielding three primary arachidonyl radicals. These radicals undergo endocyclization to form four regioisomeric prostaglandin H2-like intermediates, which are subsequently reduced to four regioisomers of the F-ring isoprostanes. Each regioisomer exists as eight racemic diastereoisomers, contributing to the structural complexity of the F2-isoprostane family [83].

F2-isoprostanes have been extensively validated as robust biomarkers of lipid peroxidation and oxidative stress across a range of diseases. Elevations in F2-IsoP levels are observed in cardiovascular diseases [84], neurodegenerative disorders such as Alzheimer’s and Parkinson’s disease [85], frailty and aging [86], diabetes mellitus [87], and even infectious diseases like COVID-19 [88]. This broad association underscores their clinical relevance in monitoring oxidative damage and disease progression.

Emerging evidence suggests that F2-isoprostanes actively participate in pathophysiological processes beyond serving as biomarkers. In bone metabolism, elevated F2-IsoP levels correlate with impaired osteoblast function and enhanced osteoclast activity, disrupting the balance between bone formation and resorption [89]. This imbalance may contribute to delayed bone healing and increased fracture risk associated with oxidative stress.

Additionally, F2-isoprostanes act as pro-inflammatory mediators, potentially exacerbating chronic inflammation at injury sites, which impairs tissue regeneration. Membrane lipid peroxidation indicated by high F2-IsoP levels can compromise membrane integrity, protein function, and DNA stability, further hampering repair mechanisms [80,81,82,83,84,90]. Despite their clear biological significance, the accurate quantification of F2-isoprostanes requires sophisticated techniques such as mass spectrometry, which offers exceptional specificity and sensitivity. The method developed by Roberts et al. achieves an accuracy of approximately 96% and precision between 5–6%, though it remains expensive and time-consuming [80]. This technique remains the gold standard for research and clinical assessments.

### 5.2. Antioxidant Biomarkers

#### 5.2.1. Glutathione

Glutathione (GSH), present in intracellular concentrations ranging from 2 to 10 mM, is the primary determinant of the cellular redox environment, predominantly existing in its biologically active reduced thiol form [91]. As a key antioxidant, GSH plays a pivotal role not only in neutralizing reactive oxygen species (ROS) but also in regulating numerous cellular processes via redox signalling and protein glutathionylation [92]. While antioxidants from dietary sources are thought to function in vivo as antioxidants and exert beneficial effects on health—including enhancing antioxidant defenses, promoting longevity, supporting cell maintenance, and facilitating DNA repair—the oral bioavailability of GSH is limited due to its degradation in the gastrointestinal tract and poor absorption [93].

The intracellular redox state is often assessed by the ratio of reduced glutathione (GSH) to oxidized glutathione disulfide (GSSG). A decrease in the GSH/GSSG ratio is a hallmark of oxidative stress, reflecting an imbalance between ROS production and antioxidant capacity. Maintaining this ratio through de novo GSH synthesis, enzymatic reduction of GSSG, and limited uptake of exogenous GSH is crucial for preserving redox homeostasis and cellular function [94,95,96].

As the major intracellular antioxidant, glutathione protects bone cells—including osteoblasts, osteoclasts, and mesenchymal stem cells (MSCs)—from oxidative damage, which is essential for efficient bone regeneration [10]. Excessive oxidative stress and prolonged inflammation can impair bone healing by damaging cellular components and disrupting signalling pathways [97]. Through its antioxidant activity, GSH mitigates this damage by scavenging ROS and regenerating other antioxidants such as vitamins C and E [98]. The sensitivity of MSCs to oxidative stress is of particular importance, as these progenitor cells are critical for osteogenesis. Studies show that glutathione preserves MSC viability and promotes their proliferation and differentiation into osteoblasts, directly influencing bone repair and remodelling. Moreover, GSH-mediated redox regulation supports the communication between osteoblasts and osteoclasts, facilitating balanced bone remodelling and mineralization—the final stage of bone healing—by creating a favorable environment for calcium and phosphate deposition [99,100].

Deficiencies in glutathione levels or disruptions in its redox cycle can lead to elevated oxidative stress, impairing osteogenic differentiation and bone matrix formation, which may contribute to pathological conditions such as osteoporosis, delayed fracture healing, and bone inflammation. Consequently, therapeutic interventions aiming to restore glutathione levels—such as supplementation with N-acetylcysteine (NAC), a GSH precursor, or agents that enhance endogenous GSH synthesis—have shown promise in improving bone healing outcomes in preclinical models [10,46,101]. Clinical and experimental studies highlight the therapeutic potential of enhancing glutathione status to counteract oxidative stress-related bone pathologies. For instance, NAC supplementation has been shown to restore GSH levels, reduce oxidative damage, and improve osteoblast function in vitro and in animal models of osteoporosis. Moreover, the interplay between glutathione and redox-sensitive transcription factors such as Nrf2 further illustrates its role in orchestrating antioxidant gene expression during bone healing [10,102,103,104].

Despite the recognized importance of glutathione, challenges remain in translating these findings to clinical practice, particularly concerning optimal dosing strategies, bioavailability, and long-term safety of GSH-enhancing supplements. Future research should focus on the development of targeted delivery systems to improve GSH bioavailability and the elucidation of precise molecular mechanisms by which glutathione modulates bone cell functions under physiological and pathological conditions.

#### 5.2.2. Superoxide Dismutase (SOD)

Superoxide dismutase (SOD) is a critical antioxidant enzyme that catalyses the dismutation of the superoxide anion (O_2_^•−^) into molecular oxygen (O_2_) and hydrogen peroxide (H_2_O_2_). By converting the highly reactive superoxide radical into the less reactive H_2_O_2_, SOD plays a vital role in reducing the formation of the highly damaging hydroxyl radical (^•^OH), thereby protecting cells from oxidative damage [40]. In humans, three isoforms of SOD have been characterized:

CuZnSOD (SOD1), a copper- and zinc-dependent isoform located primarily in the cytosol, nucleus, peroxisomes, and mitochondrial intermembrane space;

EcSOD (SOD3), a copper- and zinc-dependent isoform secreted into the extracellular space;

MnSOD (SOD2), a manganese-dependent isoform localized exclusively in the mitochondrial matrix [105,106].

Each SOD isoform requires its specific metal cofactors to maintain catalytic activity, which underscores the importance of trace elements such as copper, zinc, and manganese in diet and cellular function. SOD1 is fundamental for antioxidant defense in the cytosol and mutations in SOD1 have been implicated in neurodegenerative disorders such as amyotrophic lateral sclerosis (ALS) [107]. EcSOD, produced primarily by smooth muscle cells and secreted into extracellular spaces such as blood vessels and lungs, is vital in modulating redox balance in the extracellular matrix and regulating endothelial nitric oxide (^•^NO) bioavailability, playing a significant role in vascular homeostasis and cardiovascular diseases [108]. MnSOD, located in mitochondria, is essential for aerobic life, as mitochondria are the major source of ROS generation due to electron transport chain activity. MnSOD protects mitochondrial DNA, proteins, and lipids from oxidative injury and is critical for cell survival under oxidative stress [109].

The increased activity of SOD isoforms during bone healing reflects an adaptive response to elevated oxidative stress in regenerating tissues. Bone healing involves inflammation and enhanced metabolic activity, which are accompanied by increased production of ROS. Elevated SOD activity mitigates ROS-induced damage, thereby protecting osteoblasts, osteoclasts, and mesenchymal stem cells that are crucial for bone regeneration [110].

Several studies have demonstrated upregulation of SOD isoforms during bone repair. For example, Zhou et al. (2018) [111] showed that MnSOD expression is elevated in osteoblasts under oxidative stress, which promotes cell survival and function. Similarly, extracellular SOD (ecSOD) has been implicated in maintaining vascular integrity in the bone microenvironment, which is critical for nutrient delivery and waste removal during healing. Moreover, trace element deficiencies affecting SOD cofactors (copper, zinc, manganese) have been linked to impaired bone metabolism and delayed fracture healing. This underscores the importance of adequate nutritional support in bone regenerative therapies. In pathological conditions such as osteoporosis and chronic inflammation, oxidative stress overwhelms antioxidant defenses, including SOD systems, resulting in cellular damage and impaired healing. Therapeutic strategies aimed at enhancing SOD expression or activity, including gene therapy and pharmacological agents, have shown potential in improving bone repair by modulating oxidative stress [112,113,114].

#### 5.2.3. Glutathione Peroxidases (GPxs)

Glutathione peroxidases (GPxs) comprise a family of enzymes characterized by the presence of either selenocysteine (GPx1, GPx2, GPx3, GPx4, GPx6) or cysteine residues (GPx5, GPx7, GPx8) at their active sites. These enzymes exhibit peroxidase activity, catalysing the reduction of hydrogen peroxide (H_2_O_2_) and lipid hydroperoxides to water and corresponding alcohols, respectively [115].

The human GPx family consists of eight isoforms, each with distinct tissue distributions, substrate specificities, and physiological roles. GPx1, the most abundant isoform, primarily reduces hydrogen peroxide in the cytosol and mitochondria, playing a vital role in maintaining cellular redox homeostasis and regulating signal transduction pathways important for mitochondrial function and thiol-redox balance [116]. GPx1 protects cellular components from oxidative damage, preventing DNA mutations and exhibiting anti-carcinogenic properties (Cheff et al., 2023) [116]. Interestingly, both excessive depletion and overaccumulation of antioxidants can disrupt physiological functions; excessive antioxidant activity can lead to reductive stress, which impairs mitochondrial function, cell proliferation, and may contribute to cardiomyopathies and other pathologies [117,118]. Therefore, the balance of GPx activity is critical for maintaining redox equilibrium. The isoforms GPx2, GPx3, and GPx4 possess specialized functions relevant to tissue-specific oxidative stress responses. GPx2 is predominantly expressed in the gastrointestinal tract, where it protects the intestinal epithelium from oxidative damage and serves as a marker of gastrointestinal oxidative stress in diseases such as colorectal cancer and inflammatory bowel diseases. GPx3, secreted into plasma, serves as a systemic oxidative stress biomarker and is often reduced in chronic inflammatory conditions and cardiovascular diseases. GPx4, also known as phospholipid hydroperoxide glutathione peroxidase, is specialized in protecting membrane lipids from peroxidation, playing an essential role in preventing ferroptosis—a regulated cell death linked to lipid peroxidation—and is crucial in neurodegenerative diseases, atherosclerosis, and reproductive health [119,120]. Less abundant isoforms, such as GPx5 (epididymal), GPx6 (embryonic and nasal epithelium), GPx7, and GPx8, contribute to antioxidant defenses in specific tissues and cellular compartments, such as the endoplasmic reticulum (ER). GPx7 and GPx8 are involved in oxidative protein folding and ER redox homeostasis, with emerging roles in modulating calcium signalling and inflammatory responses [119,121].

Studies demonstrate that adequate GPx activity promotes osteogenesis by preserving the redox balance, protecting osteoblasts from oxidative damage, and facilitating differentiation and mineralization processes. Reduced GPx activity correlates with delayed fracture healing and increased susceptibility to bone diseases such as osteoporosis. Moreover, GPx activity serves as a reliable biomarker to monitor oxidative stress in bone pathologies and assess the efficacy of antioxidant therapies aimed at enhancing bone regeneration [122,123]. Therapeutic supplementation with GPx precursors or compounds that upregulate GPx expression, including selenium, has shown promise in improving fracture healing outcomes and reducing oxidative damage [122].

#### 5.2.4. Catalase (CAT)

Catalase (CAT) is implicated in diverse physiological processes such as cell proliferation signalling, apoptosis regulation, carbohydrate metabolism, and platelet activation. As a key antioxidant enzyme, catalase catalyses the decomposition of intracellular hydrogen peroxide (H_2_O_2_) into water and oxygen, thereby preventing oxidative damage to cellular components [40].

Catalase is among the most catalytically efficient enzymes known, with a single molecule converting millions of H_2_O_2_ molecules per second, highlighting its essential function in oxidative stress defense. The enzymatic mechanism involves two distinct stages. First, hydrogen peroxide oxidizes the heme iron, forming an oxyferryl intermediate known as Compound I, which contains a ferryl iron (Fe^4+^=O) and a porphyrin or protein radical. In the second stage, a second hydrogen peroxide molecule reduces Compound I back to its resting state, releasing water and oxygen. Compound I can also undergo single-electron reduction to inactive Compound II under certain conditions, affecting catalase activity [124,125,126]. Interestingly, free heme itself can catalyse hydrogen peroxide decomposition, albeit with much lower efficiency than catalase-containing proteins, explaining catalase-like activity observed in some non-catalase enzymes. Catalases can be categorized into four main types based on structure and sequence diversity: monofunctional classical catalases (Type A), bifunctional catalase-peroxidases (Type B), non-heme catalases (Type C), and proteins with low catalytic activity (Type D) [127].

Catalase plays a pivotal role in maintaining redox homeostasis by decomposing H_2_O_2_ produced as a byproduct of metabolic processes, particularly in peroxisomes. While not essential for survival under normal physiological conditions in certain cell types, catalase is crucial for adaptive responses to oxidative stress. For example, in experimental models, rats exposed to hyperoxic conditions (100% oxygen) showed increased survival when treated intravenously with liposomes containing superoxide dismutase (SOD) and catalase, underscoring catalase’s protective role [128].

Further, overexpression of catalase in transfected cells has been shown to increase resistance to oxidative stress and drug-induced cytotoxicity by efficiently decomposing H_2_O_2_ and preventing harmful ROS-mediated reactions. Conversely, catalase activity can be inhibited under conditions of chronic oxidative stress or excessive ROS, leading to impaired antioxidant defense and contributing to various pathologies including cardiovascular diseases, diabetes, neurodegenerative disorders, and cancer [126,129]. An increase in catalase activity is often observed in response to elevated ROS levels, reflecting the body’s attempt to restore redox balance during injury, inflammation, or healing processes. In the context of tissue repair and bone healing, oxidative stress is a significant factor influencing the regeneration process. Catalase, together with other antioxidant enzymes, helps mitigate ROS-induced cellular damage, thereby facilitating efficient repair and regeneration [130].

## 6. Clinical Applications and Limitations

### 6.1. The Role of Oxidative Stress Markers in Bone Healing and Fracture Monitoring—Clinical Applications and Limitations

Understanding the mechanisms of oxidative stress in the context of bone healing and monitoring fractures through the control of its markers is critically important. By enabling assessment of the bone regeneration process, early detection of potential complications is possible, ensuring effective and safe treatment. This can ultimately shorten recovery time and improve patients’ quality of life. 

The healing of facial bone fractures involves a complex interplay between inflammatory, oxidative, and regenerative pathways. Oxidative stress plays a dual role in this process. On one hand, reactive oxygen species (ROS) act as essential signaling molecules during the early inflammatory phase, promoting recruitment of immune cells and activation of osteoprogenitor cells, which are critical for initiating bone repair [52]. On the other hand, excessive or prolonged oxidative stress impairs differentiation and survival of osteoblasts and osteocytes, thereby contributing to delayed or incomplete healing [33,75].

The markers reviewed here (Table 2) —such as malondialdehyde (MDA), 4-hydroxynonenal (4-HNE), glutathione (GSH), superoxide dismutase (SOD), glutathione peroxidase (GPx), catalase (CAT), and F2-isoprostanes—offer unique insights into different aspects of oxidative stress response during bone repair. Elevated MDA and 4-HNE reflect ongoing lipid peroxidation, closely linked to tissue damage and chronic inflammation [38,45,95,105]. Conversely, antioxidant enzymes like SOD, GPx, and CAT indicate the organism’s endogenous attempt to neutralize ROS and limit oxidative injury [36,38,40].

Several studies have demonstrated a correlation between oxidative stress marker dynamics and stages of bone healing. Acute increases in oxidative markers during the inflammatory and granulation phases appear physiological and necessary for signaling and defense [26,27,28]. However, sustained elevation of oxidative stress markers during callus formation and remodeling phases correlates with pathological healing and impaired bone regeneration [46,76,89]. For example, a study by Bai et al. (2021) reported that elevated MDA and decreased antioxidant enzyme activities were significantly associated with delayed fracture healing in rat models [33]. These findings suggest that biochemical monitoring of oxidative stress markers could become an essential component of early diagnosis and personalized treatment in clinical practice. By tracking marker levels longitudinally, clinicians may distinguish between normal healing and complications such as nonunion or delayed union, allowing timely intervention [131,132].

Despite this promise, the clinical application of oxidative stress markers remains limited by several factors. Measurement variability due to different assay methods, lack of standardized reference values, and small sample sizes in many studies pose significant challenges [133]. Additionally, external confounders such as diet, smoking, medication use, and comorbidities like diabetes and cardiovascular disease can influence oxidative marker levels and must be carefully considered when interpreting results [134]. Another challenge is that oxidative stress markers are often non-specific. MDA and 4-HNE, for example, are elevated in various pathological states beyond bone injury, including cancer, cardiovascular disease, and infection [56,71]. Therefore, their use in isolation may not reliably indicate fracture status or healing progression.

The potential for antioxidant therapies to accelerate bone healing remains controversial. While some studies suggest that agents like N-acetylcysteine (NAC) or vitamin E can improve callus quality and reduce healing time [42], others report adverse effects, including impaired signaling necessary for regeneration [135,136,137]. This implies that blanket antioxidant administration may be inappropriate and calls for personalized approaches. Several researchers emphasize the need for large-scale, longitudinal clinical trials to validate specific oxidative stress markers as prognostic or diagnostic tools in bone healing [13,49,50]. The integration of oxidative stress marker monitoring with imaging techniques and clinical parameters could enhance fracture management, promoting more targeted antioxidant therapies where appropriate.

### 6.2. Future Prospects

Future research in the field of oxidative stress markers and bone healing should focus on several key areas to advance their clinical applicability and therapeutic potential. First, there is a critical need for the development and validation of standardized, reliable, and cost-effective methods for measuring oxidative stress biomarkers. Advances in point-of-care testing devices could facilitate real-time monitoring of oxidative status, allowing clinicians to make timely decisions during fracture treatment and rehabilitation.

Longitudinal studies with larger patient populations are essential to establish normative reference ranges for various markers throughout the different phases of bone healing. These studies would help distinguish physiological changes from pathological oxidative stress, enabling more accurate prognosis and personalized intervention strategies.

Integrating oxidative stress biomarker profiling with other clinical parameters and imaging techniques may improve the precision of fracture healing assessment and early identification of complications such as delayed union or nonunion. The application of machine learning and artificial intelligence could further enhance predictive models based on oxidative stress data combined with patient-specific factors.

Exploring the therapeutic modulation of oxidative stress using targeted antioxidants, nutritional supplements, or pharmacological agents holds great promise. Future clinical trials should investigate the timing, dosage, and combination of these interventions to optimize bone regeneration without interfering with the beneficial signaling roles of reactive oxygen species.

Finally, expanding research into the molecular mechanisms linking oxidative stress to bone cell function, inflammation, and extracellular matrix remodeling will provide deeper insights into the pathophysiology of impaired healing. This knowledge could lead to the identification of novel biomarkers and therapeutic targets, ultimately improving outcomes for patients with complex fractures or comorbidities that affect bone health.

### 6.3. Anitioxidant Therapeutic Strategies

- Bone healing, particularly in craniofacial fractures, is critically influenced by the delicate balance between reactive oxygen species (ROS) and the antioxidant defense system. While physiological levels of ROS contribute to angiogenesis, immune cell recruitment, and osteoblast differentiation, sustained oxidative stress disrupts these processes, leading to delayed union or non-union of fractures [33,62]. This has prompted increasing interest in antioxidant-based therapeutic strategies as potential adjuvants in maxillofacial trauma care.
- Vitamin C (ascorbic acid) is one of the most studied antioxidants in bone biology. It is an essential cofactor for collagen synthesis, thereby directly supporting extracellular matrix production and callus stability. Supplementation with 500–1000 mg/day has been shown to accelerate fracture repair, especially in populations at risk of vitamin C deficiency, such as smokers and the elderly [39,134].
- Vitamin E (α-tocopherol), a lipid-soluble antioxidant, protects cellular membranes against lipid peroxidation. Experimental studies demonstrated that oral doses of 200–400 IU/day reduce malondialdehyde (MDA) and 4-hydroxynonenal (4-HNE) levels, improving the biomechanical strength of the healing callus [66,137].
- N-acetylcysteine (NAC) replenishes intracellular glutathione (GSH), the master thiol antioxidant. Oral administration at 600–1200 mg/day prevents ROS-induced apoptosis in osteoblasts and osteocytes, while also mitigating chronic inflammation at the fracture site [33,136].
- Selenium, an essential trace element, exerts its effects mainly through glutathione peroxidase (GPx) activation. Daily supplementation of 100–200 µg of selenium (in the form of selenomethionine) has been shown to enhance mineralization of the fracture callus and reduce systemic oxidative burden [62].
- Polyphenols, such as resveratrol, quercetin, and curcumin, have also been studied for their osteoprotective roles. These compounds modulate signaling pathways such as Wnt/β-catenin and NF-κB, promoting osteoblast activity and inhibiting osteoclastogenesis [134].
- An interesting candidate is caffeine, traditionally associated with negative effects on calcium metabolism. Recent studies, however, suggest that at moderate doses (200–400 mg/day), caffeine exhibits radical scavenging activity comparable to glutathione and even higher than vitamin C, which may contribute to fracture healing [137].
- Beyond supplementation, dietary strategies are of paramount importance. Diets rich in fresh fruits, vegetables, legumes, and whole grains—exemplified by the Mediterranean diet—supply a wide range of antioxidants including vitamins, carotenoids, and polyphenols, all of which contribute to improved redox homeostasis and bone regeneration [39]. Conversely, ultraprocessed foods, alcohol, and tobacco use exacerbate oxidative stress, impair angiogenesis, and are consistently associated with delayed or impaired fracture healing [33,62].
- Taken together, antioxidant strategies should be viewed as supportive measures that complement, rather than replace, surgical and pharmacological interventions. Future clinical studies are needed to establish optimal timing, dosage, and combinations of antioxidant compounds. Personalized approaches, integrating biomarker monitoring with targeted antioxidant therapy, may significantly improve outcomes in patients with craniofacial fractures.

## 7. Conclusions

Oxidative stress markers reflect both the beneficial and detrimental roles of reactive oxygen species in bone regeneration. Evidence suggests that their monitoring could support earlier detection of impaired healing and guide individualized interventions in facial bone fractures. However, clinical application is currently limited by methodological variability, lack of reference ranges, and confounding systemic factors. Antioxidant enzymes such as SOD, GPx, and CAT appear especially promising as indicators of endogenous defense mechanisms. Progress in assay standardization and translational clinical research is essential for these biomarkers to become practical diagnostic and prognostic tools in maxillofacial trauma care.

## Figures and Tables

**Table 1:** Oxidative stress mechanisms in bone healing.

Mechanism	Positive Role (Physiological ROS)	Negative Role (Excess ROS)	Antioxidant Counteraction
Inflammation	Early recruitment of neutrophils, macrophages	Chronic inflammation, tissue damage	GSH, SOD, CAT neutralize radicals
Angiogenesis	VEGF signalling enhanced by ROS	Endothelial dysfunction, reduced perfusion	Antioxidants preserve endothelial NO
Osteoblasts	Differentiation signalling (Wnt/β-catenin, MAPK)	Apoptosis, impaired differentiation	NAC, vitamin C support osteogenesis
Osteoclasts	Normal resorption/remodelling	Excessive activation → bone loss	GPx, SOD inhibit osteoclastogenesis
Collagen synthesis	Controlled ROS stimulate ECM production	Excess ROS → impaired collagen, weak callus	Antioxidants preserve ECM integrity

**Table 2:** Comparison table of biomarkers in the context of their function, impact on bone healing and detection methods.

Marker	Function	Impact on Bone Healing	Detection Methods	Adv.	Limitat.	Ref.
MDA	End product of lipid peroxidation; indicates oxidative damage to cell membranes	Elevated levels indicate increased lipid peroxidation and tissue damage, associated with impaired bone regeneration and chronic inflammation	Thiobarbituric acid reactive substances (TBARS) assay, HPLC, spectrophotometry	Easy to measure	Low specificity	[62,65,79]
4-HNE	Reactive aldehyde formed during lipid peroxidation; modulates cell signalling and apoptosis	High 4-HNE impairs osteoblast function and promotes inflammation, potentially delaying bone healing	ELISA, HPLC, mass spectrometry	High sensitivity	Unstable, difficult to quantify	[70,71]
F2-Isoprostanes	Stable products of free radical-induced lipid peroxidation; reliable oxidative stress biomarkers	Elevated levels indicate membrane lipid damage and chronic oxidative stress, associated with impaired bone remodelling and inflammation	Mass spectrometry (GC-MS or LC-MS), ELISA	Highly specific	Mass spectrometry required	[79,80,81,82]
GSH	Major intracellular antioxidant; reduces ROS and maintains redox balance	Supports osteoblast survival and differentiation by neutralizing ROS; depletion correlates with oxidative stress and poor healing	Spectrophotometric assays, HPLC, fluorometric assays	Central redox regulator	Low oral bioavailability	[90,91,92,93,94]
SOD	Enzyme catalysing dismutation of superoxide radicals into hydrogen peroxide and oxygen	Protects bone cells from superoxide damage; increased activity indicates antioxidant defense during early healing	Activity assays, spectrophotometry, ELISA	Stable, measurable	Requires isoform-specific methods	[105,106,107,108]
GPx	Enzyme reducing hydrogen peroxide to water using GSH as substrate	Reduces H_2_O_2_ toxicity, protecting osteoblasts and promoting normal bone formation	Activity assays, spectrophotometry, ELISA	Protects osteoblasts	Selenium-dependent	[109,115,118]
CAT	Breaks down hydrogen peroxide into water and oxygen	Prevents H_2_O_2_ accumulation; protects bone cells from oxidative damage, promoting effective healing	Activity assays, spectrophotometry, ELISA	Highly efficient	Reduced by stress	[123,124,125,126]

## Data Availability

No new data were created or analyzed in this study. Data sharing is not applicable to this article.
